# Papillary thyroid carcinoma with desmoid fibromatosis: a case report and review of literature

**DOI:** 10.31053/1853.0605.v80.n3.40408

**Published:** 2023-09-29

**Authors:** Luis Agustín Ramírez Stieben, Daniel Pozzi

**Affiliations:** 1 Unidad de Tiroides y Paratiroides del Grupo Gamma Rosario Argentina; 2 Servicio de Anatomía Patológica del Grupo Gamma Rosario Argentina

**Keywords:** cáncer papilar tiroideo, desmoide, síndrome de Gardner, papillary thyroid carcinoma, desmoid, Gardner syndrome, câncer papilífero da tireoide, desmoide, síndrome de Gardner

## Abstract

Desmoid-type fibromatosis (DF) is a rare monoclonal, fibroblastic proliferation characterized by an unpredictable and variable clinical course. We present the case of a 56-year-old woman who underwent total thyroidectomy for papillary thyroid carcinoma in 2012 and who developed a cervical mass at the left laterocervical level during follow-up, raising the diagnosis of tumor recurrence. Computed tomography of the neck showed solid formations with heterogeneous contrast uptake in the right lateral region of the neck. At the level of the thoracic operculum, a second 26-mm formation was observed that medially contacted the left lateral wall of the trachea. Lateral lymphadenectomy was performed, which was incomplete. Histology showed findings consistent with desmoid-type fibromatosis. DF are slowly proliferating, non-metastatic tumors with a highly invasive capacity that are usually present in familial adenomatous polyposis (FAP)-Gardner syndrome. Our case had a history of massive colonic polyposis and first-degree relatives of colorectal cancer.

## Resumen

La fibromatosis de tipo desmoide (FD) es una rara proliferación fibroblástica monoclonal caracterizada por un curso clínico impredecible y variable. Presentamos el caso de una mujer de 56 años intervenida de tiroidectomía total por carcinoma papilar de tiroides en 2012 y que durante el seguimiento desarrolla una masa cervical a nivel laterocervical izquierdo, planteando el diagnóstico de recidiva tumoral. La tomografía computarizada de cuello demostró formaciones sólidas con captación heterogénea de contraste en la región lateral derecha del cuello. A nivel del opérculo torácico se observó una segunda formación de 26 mm que contactaba medialmente con la pared lateral izquierda de la tráquea. Se realizó una linfadenectomía lateral, que resultó incompleta. La histología mostró hallazgos compatibles con FD. La FD son tumores de proliferación lenta, no metastásicos y con una capacidad altamente invasiva que suelen estar presentes en la poliposis adenomatosa familiar (PAF)-síndrome de Gardner. Nuestro caso tenía antecedentes de poliposis colónica masiva y familiares de primer grado de cáncer colorrectal.

## Resumo

A fibromatose desmóide (FD) é uma rara proliferação fibroblástica monoclonal caracterizada por um curso clínico imprevisível e variável. Apresentamos o caso de uma mulher de 56 anos que foi submetida a tireoidectomia total por carcinoma papilífero de tireoide em 2012 e que desenvolveu uma massa cervical em nível laterocervical esquerdo durante o acompanhamento, levando ao diagnóstico de recidiva tumoral. A tomografia computadorizada do pescoço demonstrou formações sólidas com realce heterogêneo pelo meio de contraste na região lateral direita do pescoço. Ao nível do desfiladeiro torácico, observou-se uma segunda formação de 26 mm que contatava medialmente a parede lateral esquerda da traqueia. Foi realizada linfadenectomia lateral, que foi incompleta. A histologia mostrou achados compatíveis com FD. Os FD são tumores não metastáticos, de proliferação lenta, com capacidade altamente invasiva, que geralmente estão presentes na síndrome de Gardner da polipose adenomatosa familiar (PAF). Nosso caso tinha história de polipose colônica maciça e um parente de primeiro grau com câncer colorretal.

**CONCEPTOS CLAVE**

¿Qué se sabe sobre el tema?

La fibromatosis de tipo desmoide es una rara proliferación fibroblástica monoclonal caracterizada por un curso clínico impredecible y variable.

¿Qué aporta este trabajo

Nuestro caso describe cómo una infrecuente entidad, como la fibromatosis de tipo desmoide, puede simular una recurrencia de la enfermedad tumoral tiroidea.

**Divulgación**

El cáncer de tiroides es la neoplasia maligna más frecuente del sistema endocrino. Puede clasificarse en diferenciados, pobremente diferenciado e indiferenciado. Habitualmente, los tumores diferenciados (papilar y folicular) tienen buen pronóstico. Sin embargo, un porcentaje de pacientes pueden evolucionar hacia una persistencia o recurrencia de la enfermedad. Al respecto, la aparición de una lesión a nivel cervical plantea, como primera hipótesis diagnóstica, la recidiva tumoral ya que la fibromatosis de tipo desmoide es una entidad infrecuente.

## Introduction

Desmoid-type fibromatosis (DF) is a rare monoclonal, fibroblastic proliferation characterized by an unpredictable and variable clinical course. ^1^ They lack metastatic potential, they are locally invasive and cause significant morbidity and mortality. The incidence is 2–4 per million population and accounts for 0.03% of all neoplasms. ^2^

The etiology of DF is unknown. Although most DF occurs sporadically, DF is seen at increased frequency in familial adenomatous polyposis (FAP). A mutation of the *CTNNB1* encoding β-catenin is found in most sporadic DF cases and constitutional mutations of *APC* have been described as hereditary in patients with FAP. ^3^

According to its location, DF is classified into three types: intra-abdominal, abdominal, and extra-abdominal. ^4^ Extra-abdominal desmoid tumors comprise a third of desmoid tumors and usually occur in the shoulder, pelvic girdle, and limbs. Only 10-25% of extra-abdominal desmoid tumors developed in the head and neck region. The management of DF is challenging and requires a discussion at a multidisciplinary tumor board. ^5^

We present the case of a 56-year-old woman who underwent total thyroidectomy for papillary thyroid carcinoma and developed a cervical mass during follow-up.

## Clinical case

A 56-year-old female patient with a history of papillary thyroid carcinoma for which she underwent total thyroidectomy in 2012 and received I131 (cumulative dose of 450 mCi) and who evolved after five years of follow-up with the appearance of a cervical mass on the left later cervical level, raising the diagnosis of tumor recurrence. She had no allergies and had been a smoker (20 packs/year). Among other antecedents, she presented massive colonic polyposis and a first-degree family history of colorectal cancer.

After the initial surgery, the biopsy revealed a 4-cm papillary carcinoma of a predominantly classic and focally follicular pattern located in the left thyroid lobe and several scattered microscopic satellite lesions in the rest of the thyroid parenchyma with focal thyroid capsular infiltration, without exceeding it. Angiolymphatic invasion images were not observed.

The patient was under treatment with levothyroxine (LT4) at a dose of 200 μg/day and presented in the laboratory with a TSH of 0.744 μIU/ml, T4l 1.29 ng/dl, thyroglobulin 5.1 ng/ml and ultrasensitive antithyroglobulin 3.04 IU/ml (<150 IU/ml). On physical examination, she presented a left later cervical lobulated mass measuring 8 by 5 cm, with a hard elastic consistency, and partially adhered to deep planes. A neck computed tomography with intravenous contrast was requested, in which solid formations with heterogeneous contrast uptake were observed in the left lateral region of the neck, the largest of 40 mm, and located behind the primitive carotid artery and in close contact with the left jugular vein, producing an extrinsic compression

and a decrease in its caliber. Caudally to this formation, at the level of the thoracic operculum, a second 26-mm formation was zobserved that contacted medially with the left lateral wall of the trachea and, behind, with the primitive carotid artery.

**Figure. 1:**
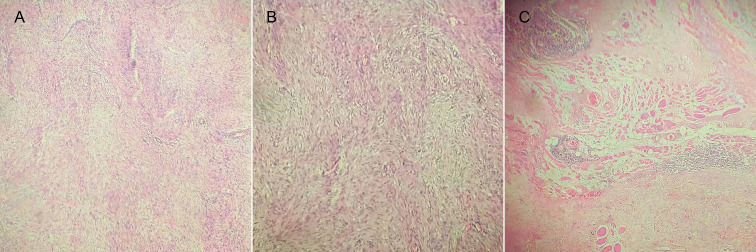
Histologic biopsy

A fine needle aspiration biopsy (FNAB) was performed which is not conclusive and a surgical resolution was decided, proceeding to a left lateral lymphadenectomy that was incomplete. Histology demonstrated the presence of important connective changes of the hypertrophic type of scar repair with the presence of inflammation. Among the fibrous tissue, areas of fusiform elements with a proliferative appearance were observed in parallel layers, without atypia and with an infiltrative arrangement on the adjacent tissues. These findings were consistent with desmoid-type fibromatosis. The eleven lymph nodes analyzed presented non-specific reactive lymphoreticular hyperplasia (Fig. 1). Immunohistochemistry was requested and reported focal AML +, negative beta catenin, negative S100, negative CK7 and negative PAX8. Based on these findings, locoregional structural recurrence of thyroid cancer is ruled out and radiotherapy (RT) of macroscopic residual tissue is considered.

A. Spindle cells with proliferative appearance in parallel layers. B. Proliferation of fibroblasts and myoblasts. C. Foci of inflammatory lymphocytic infiltrates and striated muscle fibers immersed in mesenchymal proliferation.

Likewise, considering the first-degree history of thyroid cancer, massive colonic polyposis, the presence of desmoid-type fibromatosis and thyroid cancer, the diagnosis of Gardner syndrome is considered and genetic tests and orthopantomography are requested.

## Discussion

DF are slowly proliferating, non-metastasizing tumors with a highly invasive capacity, which can be life-threatening in the case of intra-abdominal retroperitoneal localization ^1^ . DFs are poorly circumscribed masses that infiltrate the surrounding soft-tissue structures. They are formed by a myxoid stroma containing elongated and uniform spindle cells, which present rare mitotic figures. ^6^ No differences are found between extra- and intra-abdominal DF, neither in their histology nor in their clinical behavior. We present a patient with a history of thyroidectomy for thyroid cancer who, on follow-up, developed a neck mass with a histopathological diagnosis of DF. This is the fourth case of a DF after thyroidectomy.(7-9), and the third after total thyroidectomy.(7,9)

DF are usually present in familial adenomatous polyposis (FAP)-Gardner syndrome.(2,10) FAP is a hereditary cancer syndrome caused by the *APC* gene's germline mutation, which is transmitted in an autosomal dominant manner with nearly 100% penetrance. ^10^ The incidence ranges between 1:6,850 and 1:23,700 live births. ^11^ Affected people will develop hundreds to thousands of small adenomatous colonic polyps. It is characterized by an increased risk of extra-intestinal manifestations, such as osteomas, congenital hypertrophy of retinal pigment epithelium, gastric and duodenal polyps, thyroid and pancreatic cancer, adrenal cortical adenoma, hepatoblastomas, medulloblastoma or glioblastoma. ^12^ For this reason, it is recommended that patients with FAP be evaluated with an annual thyroid ultrasound from the age of 15 to 20 years. DF affect 10-15% of patients with Gardner syndrome. ^6^ Our index case had, in addition to thyroid cancer, a history of massive colonic polyposis and first-degree relatives of colorectal cancer. Although the diagnosis of Gardner syndrome was highly suspicious, ophthalmological studies and orthopantomography were normal. The patient was referred for genetic counseling to assess the genetic mutation study and the indication for prophylactic colectomy. Unfortunately, definitive diagnosis could not be reached due to the lack of availability of the mutational study of the APC gene.

Differently from sporadic DF, FAP-associated DF are mainly intra-abdominal (80% of cases) or at the abdominal wall, while extra-abdominal DF accounts for about 5% of patients. ^13^ However, our patient presented DF in the neck. In the context of a

history of thyroid cancer, this cervical mass's appearance raised the tumor recurrence diagnosis. Therefore, we performed a FNAB that was not conclusive, requiring surgery, obtaining the diagnosis of DF.

The approach to DF is primarily surgical. ^6^ Unlike other soft-tissue sarcomas where the goal of surgical resection is to achieve a microscopic negative margin (R0 resection), an R0 resection is only deemed desirable, but not a necessity in the surgery of a DF. Although positive margins after surgery have been reported as an independent prognostic factor for recurrence, guidelines prohibit a morbid surgery from achieving an R0 resection. ^14^ In combination with RT and medical therapy, the surgical treatment of DT has become more and more conservative. Therefore, RT is primarily used in the adjuvant setting when surgery leaves behind a positive margin, or when surgical resection is not possible. ^14^ In our case, total surgical resection was not possible, therefore, adjuvant radiotherapy was considered.

Finally, a rare variant of papillary carcinoma with a DF component has been reported. However, a dual tumor with a classic CPT component with malignant epithelial proliferation and another component with mesenchymal proliferation is necessary. ^15^ Our patient did not show DF in the primary tumor or in the lymph nodes evaluated later.

## Conclusions

DF are locally aggressive clonal proliferations of mesenchymal tissue. They lack metastatic potential, but their locally aggressive behavior can be associated with several complications. In the setting of FAP-syndrome, DF of the head and neck are quite rare and are often incompletely removed with grossly or microscopically residual disease. Only four cases were reported after thyroidectomy.
